# Validation of a nuclear grading system for resected stage I–IIIA, high-risk, node-negative invasive breast carcinoma in the N·SAS-BC 01 trial

**DOI:** 10.1007/s12282-022-01350-4

**Published:** 2022-04-18

**Authors:** Hitoshi Tsuda, Masafumi Kurosumi, Futoshi Akiyama, Shinji Ohno, Shigehira Saji, Norikazu Masuda, Akihiko Shimomura, Nobuaki Sato, Shintaro Takao, Shozo Ohsumi, Yutaka Tokuda, Hideo Inaji, Toru Watanabe

**Affiliations:** 1 National Defense Medical College, Saitama, Japan; 2 Kameda Medical Center, Chiba, Japan; 3 Cancer Institute of the Japanese Foundation for Cancer Research, Tokyo, Japan; 4 Cancer Institute Hospital of Japanese Foundation for Cancer Research, Tokyo, Japan; 5 Fukushima Medical University, Fukushima, Japan; 6 National Hospital Organization Osaka National Hospital, Osaka, Japan; 7 National Cancer Center Hospital, Tokyo, Japan; 8 Niigata Cancer Center Hospital, Niigata, Japan; 9 Hyogo Cancer Center, Hyogo, Japan; 10 NHO Shikoku Cancer Center, Ehime, Japan; 11 Tokai University School of Medicine, Kanagawa, Japan; 12 Kaizuka City Hospital, Osaka, Japan; 13 Hamamatsu Oncology Center, Shizuoka, Japan

**Keywords:** Histological grade, Breast cancer, Invasive ductal carcinoma, Nuclear grade, Recurrence-free survival, Overall survival

## Abstract

**Background:**

This retrospective observational study validated nuclear grading criteria developed to identify a high-risk group with recurrence rate ≥20–30% and local pathology diagnosis used in a previous multi-institutional randomized N·SAS-BC 01 trial, where the efficacy of adjuvant chemotherapy regimens was evaluated in 733 high-risk node-negative invasive breast cancer patients.

**Methods:**

Of 545 patients with long-term follow-up data (median 12.1 years), pathology slides, and local pathology diagnosis, 530 eligible patients were subjected to central pathology review (CPR) for histological type and nuclear grade (NG). Concordance in NGs was compared with local diagnosis. The 10/15-year recurrence-free survival (RFS) and overall survival (OS) rates stratified by NG and histological type were calculated.

**Results:**

Local diagnoses were invasive ductal carcinoma (IDC)-NG2, IDC-NG3, invasive lobular carcinoma (ILC), and metaplastic carcinoma (MC) in 158/327/38/7 patients, respectively. The 10/15-year RFS rates were 87.2/82.6% for IDC-NG2 and 81.8/75.0% for IDC-NG3 (*p* = 0.061), and OS rates were 95.0/92.8% for IDC-NG2 and 90.8/85.7% for IDC-NG3 (*p* = 0.042). CPR graded 485 locally diagnosed IDCs as IDC-NG1/NG2/NG3/unknown in 98/116/267/4 patients, respectively. No significant difference was found among survival curves for the three NG groups. Although the agreement level between local and CPR diagnoses was low (κ = 0.311), both diagnoses identified a patient group with a 15-year recurrence rate ≥ 20%. The 10/15-year RFS rates were 79.4/63.5% for ILC and 68.6%/unknown for MC.

**Conclusions:**

The N·SAS grading system identified a patient group with high-risk node-negative invasive breast cancer, suggesting that local diagnosis was performed efficiently in the N·SAS-BC 01 trial.

**Trial registration number**: UMIN000022571.

**Date of registration**: June 1, 2016.

**Supplementary Information:**

The online version contains supplementary material available at 10.1007/s12282-022-01350-4.

## Introduction

A variety of adjuvant chemotherapy regimens have been established for breast cancer because of its heterogeneous pathological nature, but criteria to determine optimal available therapies, tailored for each patient, are not yet fully established. A series of clinical and pathological prognostic markers have been identified and used to assist decision-making regarding the use of adjuvant therapies for breast cancer patients. Among these prognostic markers, the pathological grade of malignancy, such as histological grade, of invasive breast cancer, has been recommended for routine diagnosis [1–3].

The National Surgical Adjuvant Study for Breast Cancer (N·SAS-BC) 01 trial was a randomized clinical study that compared the efficacy and safety of oral fluoropyrimidine tegafur-uracil (UFT) with those of cyclophosphamide, methotrexate, and fluorouracil (CMF) in 733 patients with stage I to IIIA high-risk node-negative breast cancer. Because high-risk breast cancer was identified mainly by histopathological grade of malignancy of the primary tumor in the N·SAS-BC 01 trial, the N·SAS-BC Pathology Section, comprising pathologists from participating sites, established histopathological criteria including histological type, invasive size, and nuclear grade (NG) [4]. In the trial, patient eligibility was assessed at each study site because the planned number of patients was too large to use the central pathology review (CPR) system [5, 6]. Therefore, it was critical to obtain accurate and consistent diagnoses by pathologists (local diagnosis) at multiple sites, especially for NG, which is relatively subjective. To achieve accurate and consistent diagnoses prior to and during patient recruitment, the N·SAS-BC Pathology Section held periodical slide conferences to standardize the criteria for NG among pathologists and monitored interobserver agreement levels regarding diagnosis [7–9].

Many retrospective studies have reported the prognostic impact of histopathological grades, but few studies have shown the importance of grading using data from prospective clinical studies. Furthermore, in the first half of the 1990s, grading of invasive breast cancer was not popular and was not familiar to pathologists performing local diagnosis [10]; therefore, it was unclear whether pathologists at each site evaluated histological grade using standardized criteria.

Therefore, the aim of this retrospective observational study was to verify the N·SAS high-risk criteria used at 38 study sites in the N·SAS-BC 01 trial [11]. We designed this study to validate the pathology protocol part of the N·SAS-BC trial to determine whether the NG system used was appropriate as a high-risk factor and local evaluation system for NG. Upon completion of the N·SAS-BC 01 trial with over 10 years of follow-up outcomes, we evaluated the N·SAS high-risk criteria assessed by local pathologists, and correlated the respective NG data with updated long-term outcomes. The representative pathology slides of the tumors were also re-evaluated by CPR and correlated with patient outcomes.

## Materials and methods

### Ethical issues

This study was approved by an independent ethics committee at each study site and conducted in accordance with the Helsinki declaration. Informed consent was obtained from all individuals who participated in the study in principle, but in cases where patients had died for whom follow-up was censored, informed consent was waivered according to the Ethical Guidelines for Medical and Health Research Involving Human Subjects, Japan.

### Patients and study treatment in the N·SAS-BC 01 trial, and tumor samples obtained in this study

Overall, 733 patients, aged 18–75 years, with resected stage I–IIIA, node-negative, histologically confirmed invasive carcinoma that met the criteria of the high-risk group, were registered at 47 study sites from October 1996 to April 2001, and were treated with six cycles of CMF or UFT for 2 years [5, 6]. Patients with estrogen receptor (ER) and/or progesterone receptor (PgR)-positive tumors were also treated with tamoxifen for 5 years. The median follow-up time in the N·SAS-BC 01 trial was 12.1 years as of December 31, 2015 [11].

Paraffin-embedded tumor tissue blocks resected from 545 patients in the N·SAS-BC 01 trial at 38 collaborating sites were obtained. For each patient, 4-μm-thick slides of one representative cut surface of the tumor block were submitted and stained with hematoxylin and eosin (HE) at SRL Laboratories (SRL Inc., Hamura, Tokyo, Japan). This retrospective study was carried out from December 2015 to November 2018 with a data cut-off of June 30, 2018.

### Creation of criteria for high-risk node-negative breast cancer and standardization among site pathologists

High-risk criteria for node-negative invasive breast cancers used in the N·SAS-BC 01 trial were (i) invasive ductal carcinoma (IDC), NG2 or NG3 with an invasive focus of >5 mm diameter; (ii) invasive lobular carcinoma (ILC); or (iii) metaplastic carcinomas (MCs: carcinoma with squamous metaplasia, carcinoma with osseous and cartilaginous metaplasia, or spindle-cell carcinoma) [12].

NG (scores 1 to 3) was presented as the sum of the nuclear atypia score (1 to 3) and mitotic count score (1 to 3): NG1 if the sum of scores was 2 or 3, NG2 if the sum of scores was 4, and NG3 if the sum of scores was 5 or 6 [4]. For nuclear atypia, nuclei that were uniform in size and shape with unremarkable hyperchromatism were assigned a score of 1, pleomorphic nuclei and hyperchromatism with a fine-granular or reticular pattern often associated with large nucleoli were assigned a score of 3, and those falling between scores 1 and 3 were assigned a score of 2. For mitotic counts, <5 per 10 high-power fields (HPFs) was scored as 1, 5–10 per 10 HPFs was scored as 2, and ≥11 per 10 HPFs was scored as 3 after choosing the fields that appeared to contain the highest mitotic counts. Score thresholds are recommended to be adjusted according to the field diameter for the mitosis counting [12]. The most frequently used field diameter of the microscope in recent studies was 0.55 mm. However, this field diameter was not popular when patients were registered for the N·SAS-BC 01 trial, in which field diameters of 0.5, 0.53, 0.63, 0.66, and 0.68 mm were used, and therefore the score thresholds were not adjusted in this study.

In a pilot study of 230 retrospective patients prior to patient registration, 10-year recurrence rates of the high- and low-risk groups were estimated as 17–22% and 3.6–6.0%, respectively [6]. Histological evaluation of the high-risk group according to histological type, invasive size, and NG was performed by pathologists at the study sites.

Prior to and during patient registration, slide conferences presenting representative photomicrographs related to the NG method, high-risk criteria, and how to judge tumors with marginal nuclear atypia, were held periodically to familiarize the collaborating site pathologists. In five sessions of slide conferences, photomicrograph slides of 119 patients taken by 28 local pathologists were presented and the participant pathologists voted for nuclear atypia and/or the number of mitotic figures [7–9]. The results of voting and these photomicrographs edited as two volumes of a handmade pathology atlas were provided to the pathologists. Therefore, the local pathologists participated passively and actively in the creation of the grading system.

### CPR

Three pathologists (F.A., M.K., and H.T.) independently judged the HE-stained sections and scored NG according to the N·SAS-BC 01 criteria. A final NG was confirmed if two or three pathologists provided the same diagnosis. If all three pathologists gave different NGs, a median grade was adopted. A diagnosis of “special type (ILC and MC)” given by the local pathologists was confirmed by H.T. If cancer or an invasive carcinoma component was not found, a section from a different tumor area was requested.

### Endpoints

Agreement levels of NGs between local diagnosis and CPR diagnosis, and recurrence-free survival (RFS) and overall survival (OS) by IDC-NG, as well as those assessed by histological subtypes were evaluated in this study. The identification of a high-risk group using the N·SAS-BC 01 criteria was successful when the recurrence rate was ≥20–30% [4].

### Statistical analysis

Although patients in the N·SAS-BC 01 trial were treated with UFT or CMF, patient data were combined in this study regardless of treatment because RFS and OS in the two cohorts were similar: 5-year RFS rates were 88.0% in the CMF arm and 87.8% in the UFT arm (hazard ratio [HR] 0.98 [95% confidence interval [CI] 0.66–1.45], *p* = 0.92) at a median follow-up of 6.2 years (range 6.2–6.6) [5]. The number of days from randomization to the last confirmed date of no recurrence or of death from any cause was defined as RFS. The number of days from randomization to the date of death from any cause was defined as OS.

RFS and OS were estimated using the Kaplan–Meier method by IDC-NG and by other special types (ILC and MC) with HR and 95% CI, and statistical differences were tested by the log-rank test. The level of agreement of NGs between local diagnosis and CPR diagnosis was tested by Cohen’s kappa coefficient and weighted κ statistics with a 95% CI. Two-sided *p* values <0.05 were considered to indicate statistical significance. All statistical analyses were performed using SAS version 9.4 or higher (SAS Institute, Inc., Cary, NC, USA).

## Results

### Local diagnosis and CPR diagnosis of histological type and NG

Of 545 HE-stained slides, 15 contained low-risk histological type only: ductal carcinoma in situ (DCIS) component in 14 and mucinous carcinoma component in one. These 15 patients were excluded from the study and, therefore, 530 patients were included for further analyses. Although the slides from four patients contained only a small amount of IDC with a predominantly DCIS component and impossible to grade by CPR, they were included in the study population.

**Fig. 1:**
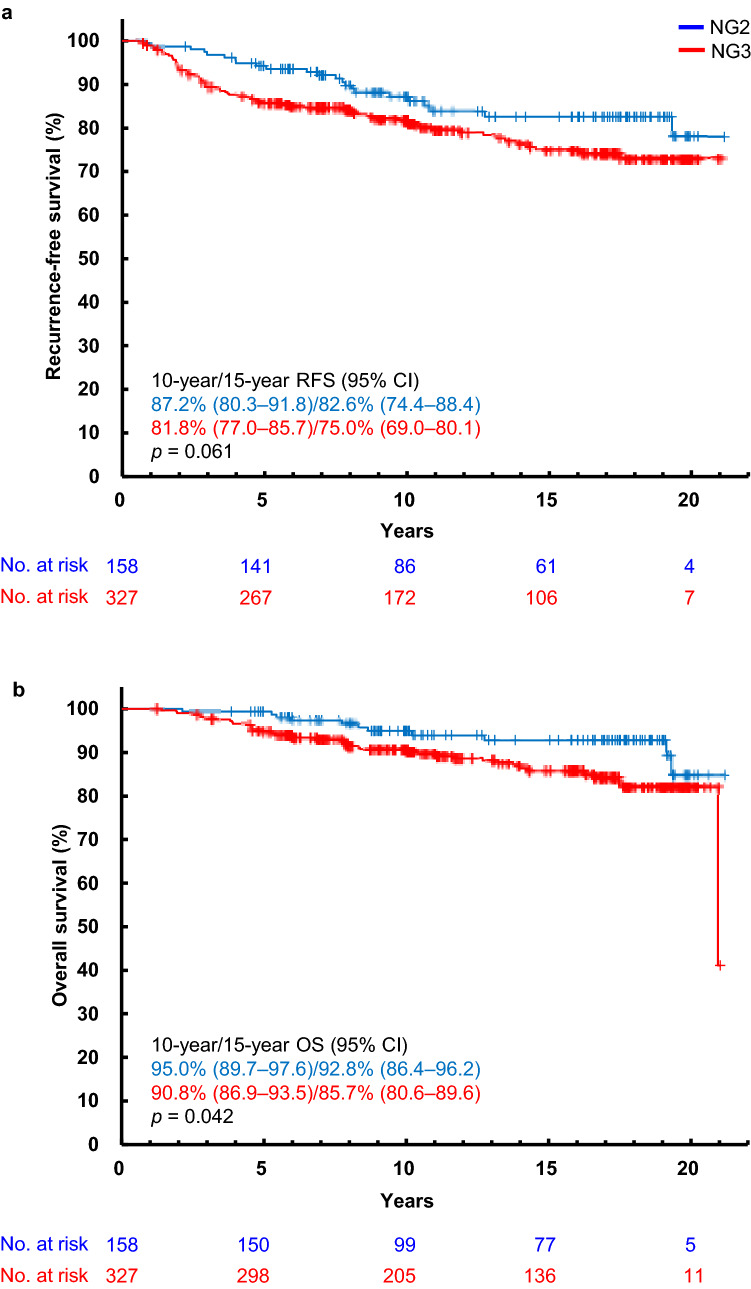
Kaplan–Meier recurrence-free survival (**a**) and overall survival (**b**) curves for patients with invasive ductal carcinoma stratified by nuclear grade assessed by local pathologists. *CI* confidence interval, *NG* nuclear grade, *OS* overall survival, *RFS* recurrence-free survival

Of 530 eligible tumors, 485 were confirmed as IDC and the remaining 45 as high-risk special types: 38 ILCs and 7 MCs (3 spindle-cell carcinomas, 3 squamous cell carcinomas, and 1 carcinoma with osseous and cartilaginous metaplasia) by local diagnosis and CPR (Table 1). The median age of the 530 patients was 53 years (range 32–75). The median invasive tumor size calculated from the case report forms was 1.95 cm (range 0.5–10.0 cm). Hormone receptor (ER and/or PgR) was positive in 366 patients (69.1%), whereas human epidermal growth factor receptor 2 (HER2) overexpression or *HER2* gene amplification was positive in 101 patients (19.1%) [11]. Of the 530 patients, the 10/15-year RFS rates were 83.1% (95% CI 79.4–86.1)/76.3% (95% CI 71.7–80.3), and the 10/15-year OS rates were 91.7% (95% CI 88.8–93.9)/86.9% (95% CI 83.1–89.9), respectively (Fig. 1, Online Resource 1).

**Table 1:** Patient baseline characteristics

Variable	Number of patients (%)
Age (years), median (range)	53 (32–75)
Tumor size (cm), median (range)	1.95 (0.5–10.0)
Histological subtype	
IDC	485 (91.5)
Special type	45 (8.5)
Invasive lobular carcinoma	38 (7.2)
Metaplastic carcinoma	7 (1.3)
Nuclear grade (local diagnosis)	
IDC Grade 2	158 (29.8)
IDC Grade 3	327 (61.7)
Special type	45 (8.5)
Nuclear grade (central pathology review)	
IDC Grade 1	98 (18.5)
IDC Grade 2	116 (21.9)
IDC Grade 3	267 (50.4)
Unknown	4 (0.8)
Special type	45 (8.5)
ER	
Negative	168 (31.7)
Positive	358 (67.5)
Unknown	4 (0.8)
PgR	
Negative	229 (43.2)
Positive	297 (56.0)
Unknown	4 (0.8)
Hormone receptor status	
ER− and PgR−	160 (30.2)
ER+ or PgR+, or both	366 (69.1)
Unknown	4 (0.8)
HER2	
Negative	420 (79.2)
Positive	101 (19.1)
Unknown	9 (1.7)
Subtype	
Hormone receptor+ and HER2+	55 (10.4)
Hormone receptor− and HER2+	45 (8.5)
Hormone receptor+ and HER2−	306 (57.7)
Hormone receptor− and HER2−	114 (21.5)
Unknown	10 (1.9)
Ki-67, median (range)	24.05 (0.0–97.2)
<20%	229 (43.2)
≥20%	301 (56.8)

Values are presented as number (%) unless otherwise indicated

*ER* estrogen receptor, *HER2* human epidermal growth factor receptor 2, *IDC* invasive ductal carcinoma, *PgR* progesterone receptor

### RFS and OS by NG in patients with IDC

Of the 485 IDCs, 158 and 327 patients were graded as NG2 and NG3, respectively by local diagnosis (Table 1). The 10/15-year RFS rates of the 158 NG2 patients were 87.2% (95% CI 80.3–91.8)/82.6% (95% CI 74.4–88.4), and those of 327 NG3 patients were 81.8% (95% CI 77.0–85.7)/75.0% (95% CI 69.0–80.1), respectively. Although no significant difference was found, patients with NG2 tended to have higher RFS rates than those with NG3 (*p* = 0.061) (Fig. 1a). The 10/15-year OS rates of the NG2 patients were 95.0% (95% CI 89.7–97.6)/92.8% (95% CI 86.4–96.2), and those of the NG3 patients were 90.8% (95% CI 86.9–93.5)/85.7% (95% CI 80.6–89.6), respectively. By local diagnosis, OS curves were significantly different between the NG2 and NG3 groups (*p* = 0.042) (Fig. 1b).

**Fig. 2:**
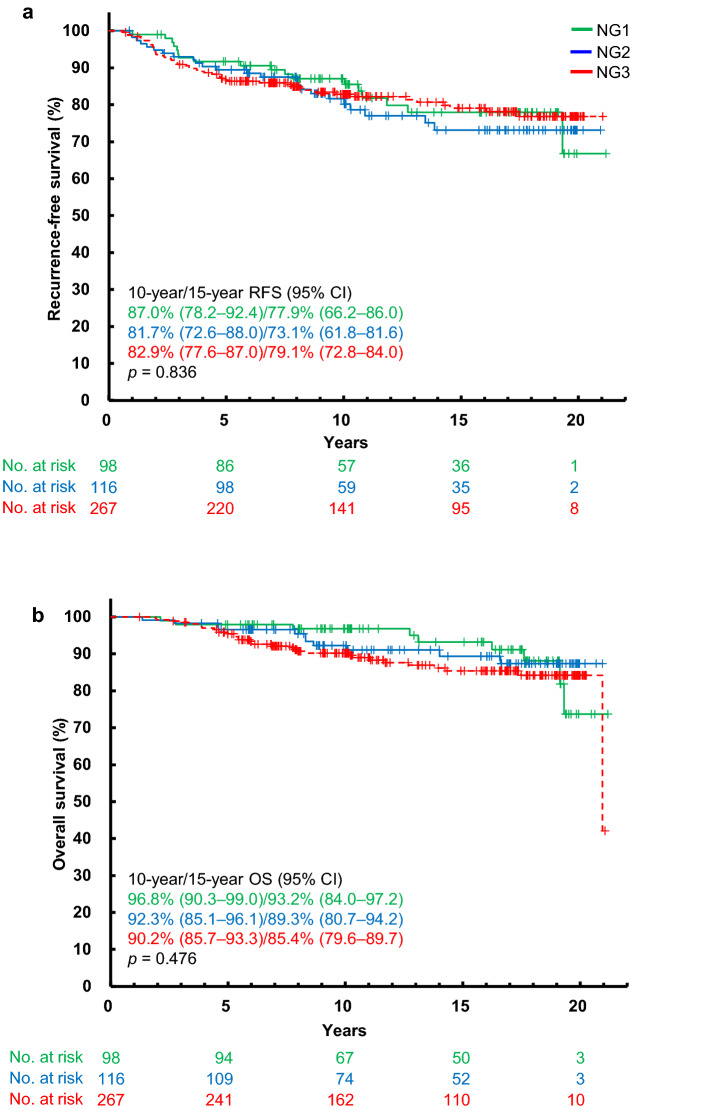
Kaplan–Meier recurrence-free survival (**a**) and overall survival (**b**) curves for patients with invasive ductal carcinoma stratified by nuclear grade assessed by central pathology review. *CI* confidence interval, *NG* nuclear grade, *OS* overall survival, *RFS* recurrence-free survival

CPR diagnoses of the 485 IDCs were NG1/NG2/NG3/unknown in 98/116/267/4 patients, respectively (Table 1). For the four patients of unknown diagnosis, grading was impossible by CPR because the invasive component was too small. The 10/15-year RFS rates of the 116 NG2 patients were 81.7% (95% CI 72.6–88.0)/73.1% (95% CI 61.8–81.6), and those of the 267 NG3 patients were 82.9% (95% CI 77.6–87.0)/79.1% (95% CI 72.8–84.0), respectively. Likewise, the 10/15-year RFS rates of the 98 NG1 patients were 87.0% (95% CI 78.2–92.4)/77.9% (95% CI 66.2–86.0), respectively. No significant difference in RFS was found between the three NGs (*p* = 0.836) (Fig. 2a). The 10/15-year OS rates of the 116 NG2 patients were 92.3% (95% CI 85.1–96.1)/89.3% (95% CI 80.7–94.2), and those of the 267 NG3 patients were 90.2% (95% CI 85.7–93.3)/85.4% (95% CI 79.6–89.7), respectively. Likewise, the 10/15-year OS rates of the 98 NG1 patients were 96.8% (95% CI 90.3–99.0)/93.2% (95% CI 84.0–97.2), respectively. Although the OS rates tended to be inversely lower in accordance with the NG, no significant difference was found between the curves of the three NG groups (*p* = 0.476) (Fig. 2b).

### Agreement level in NG between local diagnosis and CPR diagnosis

The agreement level for the NG of 481 IDCs evaluated locally and by CPR was low with a κ coefficient of 0.240 (95% CI 0.180–0.301) and a weighted κ coefficient of 0.311 (0.256–0.366). Furthermore, 71 (45.2%) of 157 NG2 patients and 27 (8.3%) of 324 NG3 patients determined by local diagnosis were classified as NG1 by CPR (Table 2).

**Table 2:** Nuclear grades assessed for invasive ductal carcinomas by local diagnosis and central pathology review

Local diagnosis	Central pathology review			Total
	Grade 1	Grade 2	Grade 3	
Grade 2	71 (45.2)	50 (31.8)	36 (22.9)	157 (100.0)
Grade 3	27 (8.3)	66 (20.4)	231 (71.3)	324 (100.0)
Total	98	116	267	481

Kappa value = 0.240 (95% confidence interval: 0.180–0.201)

Weighted kappa value = 0.311 (95% confidence interval: 0.256–0.366)

Values are presented as number (%)

### RFS and OS in patients with ILC and MC

**Fig. 3:**
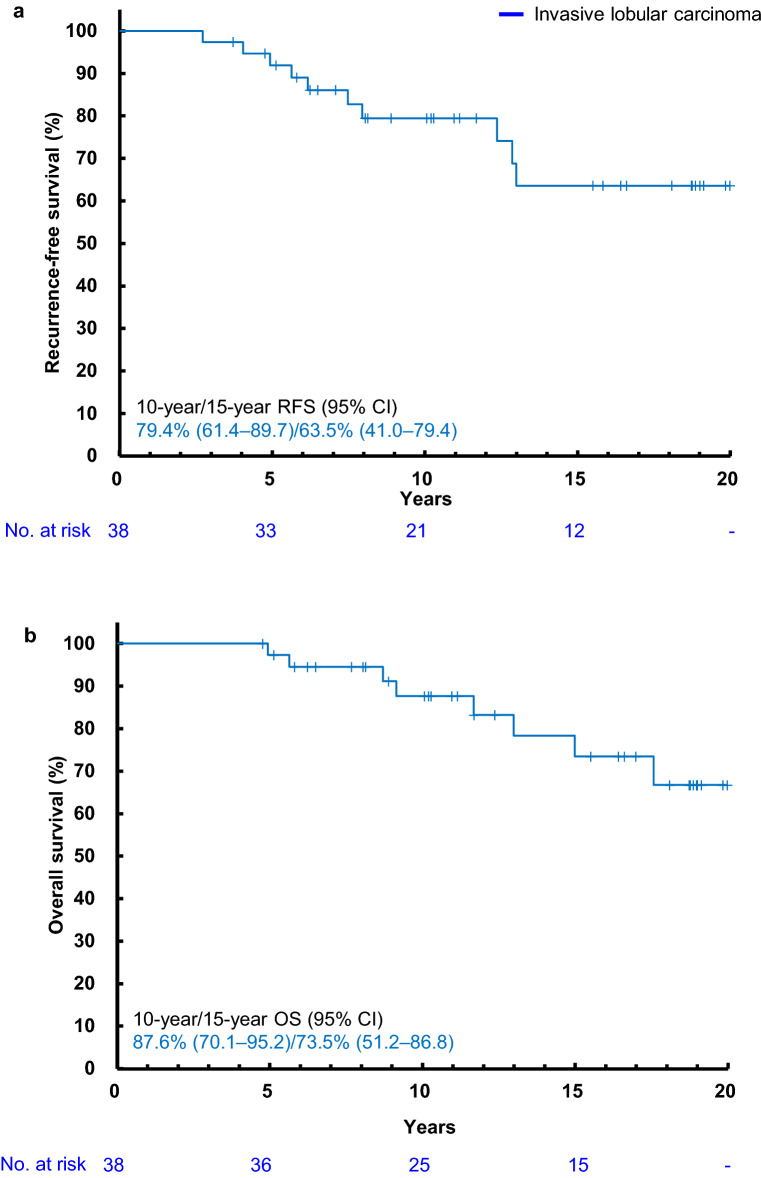
Kaplan–Meier recurrence-free survival (**a**) and overall survival (**b**) curve for patients with invasive lobular carcinoma. *CI* confidence interval, *OS* overall survival, *RFS* recurrence-free survival

Regarding the special types, the 10/15-year RFS rates were 79.4% (95% CI 61.4–89.7)/63.5% (95% CI 41.0–79.4) in ILC patients (Fig. 3) and 68.6% (95% CI 21.3–91.2)/unknown in MC patients, respectively. The 10/15-year OS rates were 87.6% (95% CI 70.1–95.2)/73.5% (95% CI 51.2–86.8) in ILC patients (Fig. 3) and 85.7% (95% CI 33.4–97.9)/85.7% (95% CI 33.4–97.9) in MC patients, respectively.

## Discussion

The N·SAS grading system was developed to identify a high-risk patient group with a recurrence rate of ≥20–30% from all patients with node-negative breast cancer [4]. The present validation study demonstrated that the 15-year recurrence rate of the NG3 group exceeded 20% by local diagnosis, and all NG1, NG2, and NG3 groups showed >20% of the 15-year recurrence rates by CPR diagnosis. In high-risk special types, ILC also showed >20% of the 10/15-year recurrence rates and >20% of the 15-year mortality rates, and MCs showed >30% of 10-year recurrence rate. These results indicate that the N·SAS grading system for the identification of high-risk groups in the N·SAS-BC 01 trial appropriately identified higher risk node-negative breast cancer groups.

The clinical outcomes of histopathologically eligible and non-eligible patient groups were not directly compared in this study. Nevertheless, we approximated the survival data of patients with node-negative breast cancer of NG1 from a single institute as those of the non-eligible group [13]. Ono et al. described the RFS and breast cancer-specific survival, instead of the OS, of 530 patients who received surgical therapy for node-negative invasive breast cancer between 1996 and 2000. For the primary tumors of all these patients, eligibility for the N·SAS-BC 01 trial was evaluated histologically. In that study, special histological types, such as ILC and MCs, as well as IDC were classified as NG1, NG2, or NG3, and non-invasive carcinoma was included. The study demonstrated that the 10-year recurrence and breast cancer-specific mortality rates of the NG2 group were 11.9 and 3.6%, respectively, similar to those by local diagnosis in the present study (12.8 and 5.0%, respectively). Furthermore, the 10-year recurrence and breast cancer-specific mortality rates of the NG3 group were 20.6 and 12.9%, respectively, similar to those by local diagnosis in the present study (18.2 and 9.2%, respectively). From these comparisons, the survival data of the NG2 and NG3 patient groups appear to be similar between the two studies. Likewise, the 10-year recurrence and breast cancer-specific mortality rates of the NG1 group reported by Ono et al. were 7.0 and 0.8%, respectively, and these rates were speculated to be close to the clinical outcomes of the non-eligible group in the N·SAS-BC 01 trial. These data indirectly suggest a much better clinical outcome for non-eligible patients compared with eligible patients.

It has been repeatedly argued that CPR is important for guaranteeing the quality of clinical trials [14, 15]. However, in the N·SAS-BC 01 trial, we used local pathology diagnosis because of the large number of patients (enrolled at 48 study sites) to be evaluated, which was unrealistic using CPR alone. To standardize the criteria of NG among local pathologists, the N·SAS-BC Pathology Section conducted the various activities described above [7–9]. The present study verified the local evaluation system with reference to the long-term clinical outcome of the patients, and showed that the local pathology diagnosis system used in the N·SAS-BC 01 trial worked efficiently.

The CPR diagnoses for 530 breast cancers in the current study classified a substantial proportion of locally diagnosed high-grade IDCs as NG1; however, the prognosis of these NG1 was very similar to NG2/NG3. Although the 15-year RFS and OS rates based on CPR were similar to those based on local pathology diagnosis, no significant survival difference was found between these three NG groups. We initially expected that detectability of the high-risk group would be lower by local diagnosis than by CPR diagnosis, and that CPR might identify a group of NG1 cases that were erroneously classified as NG2 or NG3 but who showed excellent clinical outcomes. However, in contrast to expectations, local pathologists correctly identified the high-risk patient group, and the clinical outcomes of patients with NG3 tumors were significantly or nearly significantly worse than those of patients with NG2 tumors. Furthermore, CPR identified a number of NG1 cases in locally diagnosed NG2 or NG3 cases; however, they did not observe an excellent clinical outcome for these NG1 cases or reveal a difference in the survival curves among three NG patient groups.

This tendency was the same for histological grading. Histological grade was not determined by local pathologists in the N·SAS-BC trial but was assessed by CPR in parallel with NG. Histological grade according to the World Health Organization recommendation, 4th edition [1], by CPR could not stratify patients into different clinical outcomes among patient groups with Grade I, II, and III tumors, and the outcome tended to be worse in patients with Grade II tumors than in patients with Grade III tumors (data not shown).

From these results, we considered that the poor prognostic value of CPR-based NG was not caused by a defect in NG itself but by the CPR system used. One possible reason for the failure of prognostication by NG may be attributed to intratumor heterogeneity. Only one slide per patient was submitted for CPR, and therefore, NG might have been evaluated for the lowest or lower grade portion of the tumor. Such sampling bias might have caused the downgrading of tumors from NG2 or NG3 to NG1. Nonetheless, the success of the NG system conducted by local pathologists strongly suggests that NG is a predictor of the long-term outcomes in node-negative high-risk breast cancer.

Furthermore, the agreement level between local diagnosis and CPR diagnosis was low, which might be explained by the imbalanced case distribution related to kappa statistics, intratumor heterogeneity, and the effect of the intermediate nature of NG in cases. First, in the present kappa analysis, NG1 cases determined by local diagnosis were not included in the model. Most locally diagnosed NG1 cases should have been diagnosed as NG1 by CPR, but these values were not included in the analyses, and this imbalanced 3 × 2 model appeared to have caused the apparent low kappa values. Second, as described above, sampling bias for tumors with intratumor heterogeneity might have caused the downgrading of some cases. Another possibility might be the presence of tumors with an intermediate degree of atypia. Such tumors with an intermediate nature between NG1 and NG2 or NG2 and NG3 would result in a low level of interobserver agreement [7].

The prognosis of ILC was similar to that of IDC in general, and a histologically high-risk subset of ILC based on the Ki-67 and/or histological grade, and ER status was reported previously [16, 17]. We did not perform this type of grading for ILC because, currently, a diagnosis of ILC is almost selectively given for classical type ILC alone in Japan, and is usually classified as NG1. Therefore, this suggests the necessity of a risk classification for patients with node-negative ILC other than the NG system. It is well known that MCs have a high risk of recurrence and mortality [18], and the present study confirmed the worse clinical outcome for patients with MC.

Study limitations included the small number of events related to the good prognosis of patients, and the small sample size for special types. Another limitation is that we could not conduct CPR for locally non-eligible node-negative invasive carcinomas. The examination of these non-eligible patients might further prove the validity of the N·SAS-BC grading system for the identification of high-risk groups.

In conclusion, the N·SAS grading system identified patients with high-risk node-negative invasive breast cancer, and the local pathology diagnosis system including interobserver standardization activities of the NG criteria was efficient in the trial. Because local pathology diagnosis at each study site can rapidly provide diagnosis of a larger number of patients compared with CPR, the introduction of local pathology diagnosis should be the method of choice for large-scale multi-institutional studies if standardization of pathology criteria can be achieved among participating pathologists.

## Supplementary Information

Below is the link to the electronic supplementary material.

Supplementary file1 (PDF 99 KB)

## Data Availability

The datasets used and/or analyzed during the current study are available from the corresponding author on reasonable request.
