# Correction to: Validation of a nuclear grading system for resected stage I–IIIA, high-risk, node-negative invasive breast carcinoma in the N·SAS-BC 01 trial

**DOI:** 10.1007/s12282-022-01367-9

**Published:** 2022-04-30

**Authors:** Hitoshi Tsuda, Masafumi Kurosumi, Futoshi Akiyama, Shinji Ohno, Shigehira Saji, Norikazu Masuda, Akihiko Shimomura, Nobuaki Sato, Shintaro Takao, Shozo Ohsumi, Yutaka Tokuda, Hideo Inaji, Toru Watanabe

**Affiliations:** 1 National Defense Medical College, Saitama, Japan; 2 Kameda Medical Center, Chiba, Japan; 3 Cancer Institute of the Japanese Foundation for Cancer Research, Tokyo, Japan; 4 Cancer Institute Hospital of Japanese Foundation for Cancer Research, Tokyo, Japan; 5 Fukushima Medical University, Fukushima, Japan; 6 Nagoya University Graduate School of Medicine, Nagoya, Japan; 7 National Cancer Center Hospital, Tokyo, Japan; 8 Niigata Cancer Center Hospital, Niigata, Japan; 9 Hyogo Cancer Center, Hyogo, Japan; 10 NHO Shikoku Cancer Center, Ehime, Japan; 11 Tokai University School of Medicine, Kanagawa, Japan; 12 Kaizuka City Hospital, Osaka, Japan; 13 Hamamatsu Oncology Center, Shizuoka, Japan

## Correction to: Breast Cancer 10.1007/s12282-022-01350-4

In the original publication of the article, the affiliation of the co-author, Norikazu Masuda, should be changed from “National Hospital Organization Osaka National Hospital, Osaka, Japan” to “Nagoya University Graduate School of Medicine, Nagoya, Japan”.

