# Supplementary material for: Validation of a nuclear grading system for resected stage I–IIIA, high-risk, node-negative invasive breast carcinoma in the N·SAS-BC 01 trial
Source: Breast Cancer. 2022 Apr 18;29(4):720–9. doi: 10.1007/s12282-022-01350-4 (PMC9225977; doi:10.1007/s12282-022-01350-4)
Supplement: Supplementary file 1 — Supplementary file1 (PDF 99 KB) [file 12282_2022_1350_MOESM1_ESM.pdf]

Journal: Breast Cancer
Validation of a nuclear grading system for resected stage I-IIIA, high-risk, node-negative invasive breast carcinoma in the N･SAS-BC-01 trial
Hitoshi Tsuda[1] • Masafumi Kurosumi[2] • Futoshi Akiyama[3] • Shinji Ohno[4] • Shigehira Saji[5] • Norikazu Masuda[6] • Akihiko Shimomura[7] • Nobuaki Sato[8] • Shintaro Takao[9] • Shozo Ohsumi[10] • Yutaka Tokuda[11] • Hideo Inaji[12] • Toru Watanabe[13]

[1]National Defense Medical College, Saitama, Japan; [2]Kameda Medical Center, Chiba, Japan; [3]Cancer Institute of the Japanese Foundation for Cancer Research, Tokyo, Japan; [4]Cancer Institute Hospital of Japanese Foundation for Cancer Research, Tokyo, Japan; [5]Fukushima Medical University, Fukushima, Japan; [6]National Hospital Organization Osaka National Hospital, Osaka, Japan; [7]National Cancer Center Hospital, Tokyo, Japan; [8]Niigata Cancer Center Hospital, Niigata, Japan; [9]Hyogo Cancer Center, Hyogo, Japan; [10]NHO Shikoku Cancer Center, Ehime, Japan; [11]Tokai University School of Medicine, Kanagawa, Japan; [12]Kaizuka City Hospital, Osaka, Japan; [13]Hamamatsu Oncology Center, Shizuoka, Japan

Corresponding author e-mail: htsuda@ndmc.ac.jp

a

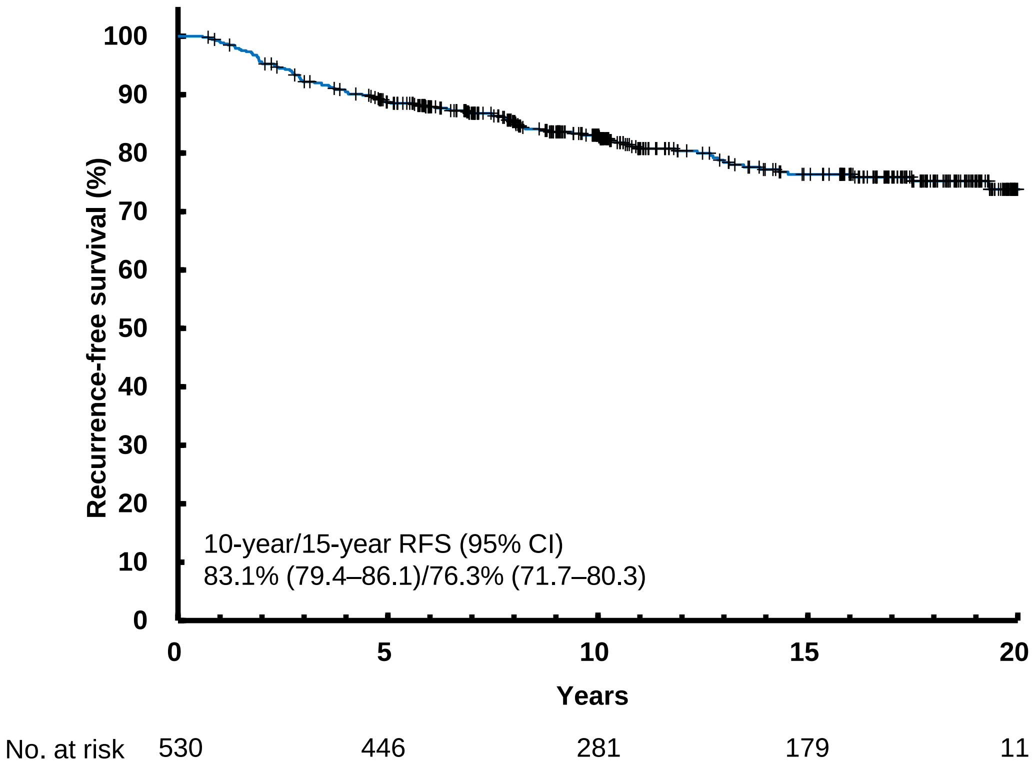

b

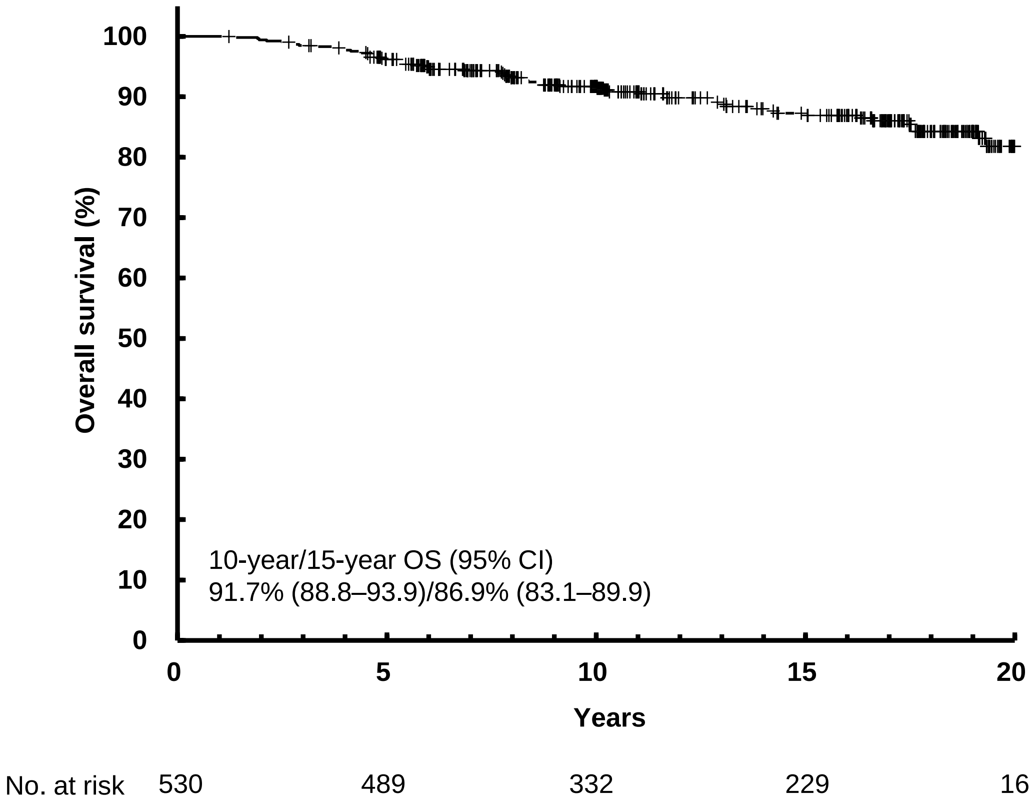

Online resource 1. Kaplan–Meier recurrence-free survival (a) and overall survival (b) curves for all patients
CI, confidence interval; OS, overall survival; RFS, recurrence-free survival
